# Serum Lactic Acid Following Aneurysmal Subarachnoid Hemorrhage Is a Marker of Disease Severity but Is Not Associated With Hospital Outcomes

**DOI:** 10.3389/fneur.2018.00593

**Published:** 2018-07-23

**Authors:** Roy A. Poblete, Steven Yong Cen, Ling Zheng, Benjamin A. Emanuel

**Affiliations:** Department of Neurology, Keck School of Medicine, University of Southern California, Los Angeles, CA, United States

**Keywords:** subarachnoid hemorrhage, aneurysm, lactic acid, medical complications, vasospasm, delayed cerebral ischemia

## Abstract

**Background:** Following aneurysmal subarachnoid hemorrhage, peripherally-drawn lactic acid has been associated with poor outcomes; however, its clinical significance is unknown. We investigated admission factors and patient outcomes associated with serum lactic acid in this population.

**Methods:** This was a retrospective observational study of 105 consecutive patients with serum lactate collected within 24 h of admission. Primary objectives were to determine the incidence of admission lactic acidemia, and factors positively and negatively associated with lactate levels. We also sought to determine if admission lactic acidemia was associated with patient outcomes, including vasospasm, delayed cerebral ischemia, mortality, and discharge disposition.

**Results:** Admission serum lactic acid was elevated in 56 patients (53% of the cohort). Levels were positively associated with Hunt & Hess and modified Fisher grade, glucose, troponin I and white blood cell counts, and negatively associated with GCS and ventilator-free days. Admission lactate was not associated with the development of vasospasm or delayed cerebral ischemia. Patients with elevated lactic acid more often died during hospitalization, and less often were discharged home. After adjusting for other predictors of poor outcome, the adjusted odds of inpatient mortality (OR 0.97, 95% CI 0.79–1.20; *p* = 0.80) and discharge to home (OR 1.00, 95% CI 0.80–1.26; *p* = 0.97) was not associated with admission lactic acid.

**Conclusions:** Early serum lactic acid elevation is common following aneurysmal subarachnoid hemorrhage and is associated with the clinical and radiographic grade of hemorrhage. Levels did not independently predict short-term outcomes when adjusted for established predictors of poor outcome. Further study is needed to determine the clinical significance of peripherally-drawn lactic acid in aneurysmal subarachnoid hemorrhage.

## Introduction

The pathophysiology of aneurysmal subarachnoid hemorrhage (aSAH) is complex. Following rupture, several mechanisms lead to secondary brain injury, including systemic inflammation, sympathetic overactivity, dysautoregulation, microcirculatory failure and ischemia. Metabolic crisis after aSAH predicts poor patient outcomes (1–4) and may represent the final common pathway leading to neuronal damage.

There is growing interest in lactate as a marker of metabolic crisis and disease severity following neurologic injury. In aSAH, lactic acid (LA) has been largely investigated using jugular bulb and microdialysis catheters, where elevated cerebral lactate portends worse outcomes (1, 2, 4–6). It is further possible to differentiate between “good” and “bad” lactate. As part of the stress response, cerebral hyperglycosis promotes lactate production and metabolism as an alternate fuel source to glucose; however, when accompanied by low oxygen tissue tension, elevated LA and lactate:pyruvate ratio may indicate true metabolic crisis (5–7).

In comparison, the clinical importance of serum lactate in aSAH is unknown. Two recent retrospective cohort studies have suggested that elevated admission serum LA is independently associated with inpatient mortality, poor outcome and delayed cerebral ischemia (DCI) (8, 9). The reason for serum LA elevation is unclear and could result from cerebral processes, primary systemic ones, or both. The aim of this study was to further characterize serum LA in the aSAH population by identifying associations between lactate and other admission variables. The association between LA and hospital outcomes was also investigated. If serum lactate is a reliable marker for pathologic systemic disease or a predictor of patient outcomes, it may serve as an important clinical target in reducing secondary brain injury in aSAH. To our knowledge, no previous study has as comprehensively described these relationships.

## Materials and methods

### Study population

Between August 2012 and April 2016, 378 adult patients with a primary diagnosis of suspected aSAH were retrospectively identified using International Classification of Disease 9th and 10th revision codes. Patients were excluded if aSAH was not the primary reason for admission, a non-aneurysmal cause of SAH was found, if the initial exam suggested brain death, if they had end-stage renal disease, if an active bacterial infection was known and if serum LA was not drawn within 24 h of admission. A total of 105 aSAH patients were included in the primary analysis. No definitive bleeding source was found in 10 patients. Two patients died before angiographic imaging. In the remaining 8 patients, no ruptured aneurysm was found on CT or digital subtraction angiogram (CTA or DSA). Bleeding patterns in this group were suggestive of aneurysm rather than perimesencephalic hemorrhage, and 8 of 10 patients required external ventricular drain (EVD) placement. All patients were admitted to the neuroscience intensive care unit (ICU) at our institution, a tertiary care center, directly from a transferring hospital. Transport typically occurred on the same hospital day as presentation. Comprehensive medical records from the transferring center accompany the patient and are integrated in to the electronic medical record (EMR). As our standard practice, patients are monitored in the ICU by a team of neurointensivists, neurosurgeons and critical care physicians for at least 10 days. Pharmacologic treatment includes nimodipine and statin therapy as tolerated, and blood pressure augmentation if angiographic or clinical vasospasm is detected.

### Data collection

Patient data was retrospectively collected from the EMR by a single researcher. For admission variables, first recorded values were obtained from the transferring hospital, or from our institution's EMR if not previously captured. Admission characteristics collected include patient demographics and co-morbidities, clinical and radiographic grades of aSAH, patient presentation, mechanical ventilation status, and standard serum studies. In our ICU, serum LA is routinely ordered given the prevalence of lactic acidemia we encounter after aSAH. Patient outcomes collected include ICU and hospital days, EVD days, complications that include vasospasm and DCI, in-hospital mortality, and discharge disposition. Data collection and management protocols were approved by the hospital Institutional Review Board, who waived the need for informed consent.

### Study objectives

The primary objective of this study was to determine the incidence of admission lactic acidemia acutely following aneurysmal subarachnoid hemorrhage, and factors positively and negatively associated with peripherally-drawn lactic acid levels. Potentially associated factors investigated include several admission characteristics, in-hospital complications, and discharge outcomes that could reliably collected from the EMR.

### Definitions

Serum LA was considered elevated when >2.2 mmol/L based on laboratory normals. Vasospasm was diagnosed using a combination of transcranial Doppler ultrasound (typically anterior circulation mean flow velocities >120 cm/s with a Lindegaard Ratio >3, vertebral or basilar artery mean flow velocities >80 cm/s), CTA or DSA. DCI was defined by persistent focal neurologic deficit attributable to vasospasm, or delayed infarct seen on CT or MRI not directly related to procedures or surgery. On transthoracic echocardiogram, an ejection fraction (EF) ≤ 40% was considered reduced, 41–70% was considered normal, and >70% was considered hyperdynamic. Fever was defined as a core temperature ≥38°C. No patients were hypothermic on presentation.

### Statistical analysis

Normality was examined for all variables of interest using histogram and D′Agostino′s *K*^2^ tests. Due to non-normal distribution for some measurements, Spearman correlation was used to examine the associations between LA and continuous admission variables. Chi-square test was used to determine differences in most dichotomized outcome variables between those with an elevated admission LA compared to those with normal levels (≤ 2.2 mmol/L). When small cells of < 5 were present, Fisher′s exact test was employed. Between patient categories, differences in most continuous outcome variables were analyzed using Wilcoxon rank sum test due to non-normal distribution. For continuous variables with normal distribution, independent *t*-test was used. Unadjusted and adjusted rate ratios for in-hospital mortality and discharge disposition home were calculated using Poisson regression model to determine the association with lactic acid levels. Adjusted rate ratios were controlled for age, GCS, HH score, and DCI, based on consistent predictors of outcome in aSAH (10). All statistical testing was two-sided and performed at a 5% level of significance using SAS version 9.4 (SAS Institute, Inc., Cary, NC, USA).

## Results

A total of 105 consecutive adult patients with suspected aSAH and admission serum LA drawn were included in primary analysis. Levels >2.2 mmol/L were present in 56 patients (53% of the population), with a mean of 2.9 1mmol/L. Baseline characteristics in patients with elevated LA vs.

those with normal LA are shown in Table 1. There were no differences in age, gender or co-morbidities between groups, apart from pre-existing hypertension. Patients with elevated LA were significantly more likely to be non-white, have been found down, and have poorer Glasgow Coma Scale (GCS), HH Scale, and modified Fisher Scale scores. On transthoracic echocardiogram, wall-motion abnormalities (WMAs) were more commonly found with elevated LA; however, systolic function was not different. Groups had similar hemoglobin, bicarbonate, anion gap and creatinine. Those with elevated LA had significantly higher white blood cell counts (WBCs), serum glucose, and troponin I.

**Table 1:** Baseline characteristics for 105 suspected aneurysmal subarachnoid hemorrhage patients with admission serum lactic acid drawn.

**Variable**	**Lactate elevated (*n* = 56) No. (%)**	**Lactate normal (*n* = 49) No. (%)**	***p*-value**
Age, mean ± SD	59 ± 13	60 ± 15	0.87
Gender			0.64
Male	17 (30)	17 (35)	
Female	39 (70)	32 (65)	
Race			**0.02**
White	5 (9)	13 (26)	
Hispanic	32 (57)	17 (35)	
Other	19 (34)	19 (39)	
Glasgow Coma Scale Score, median (IQR)	7 (6–13)	14 (10–15)	<**0.01**
Hunt & Hess Grade, median (IQR)	4 (3–5)	2 (2–3)	<**0.01**
Modified Fisher Grade, median (IQR)	4 (4–4)	4 (3–4)	<**0.01**
Found Down			**0.01**
Yes	16 (29)	4 (8)	
No	40 (71)	45 (92)	
Fever			
Yes	2 (4)	1 (2)	1.0
No	54 (96)	48 (98)	
Circulation			0.25
Anterior	44 (79)	37 (76)	
Posterior	9 (16)	5 (10)	
None^a^	3 (5)	7 (14)	
Coronary Artery Disease			1.0
Yes	3 (5)	2 (4)	
No	53 (95)	47 (96)	
Heart Failure		2 (100)	0.22
Yes	0 (0)	2 (4)	
No	56 (100)	47 (96)	
Ejection Fraction^b^			0.30
normal	38 (72)	36 (75)	
reduced	5 (9)	1 (2)	
hyperdynamic	10 (19)	11 (23)	
Wall-Motion Abnormalities^b^			**0.04**
Yes	13 (25)	4 (8)	
No	40 (75)	44 (92)	
Hypertension			**0.04**
Yes	0 (0)	4 (8)	
No	56 (100)	45 (92)	
Diabetes Mellitus			0.34
Yes	12 (21)	7 (14)	
No	44 (79)	42 (86)	
Chronic Renal Disease			0.68
Yes	4 (7)	2 (4)	
No	52 (93)	47 (96)	
WBC, 10^3^ cells/μL mean ± SD	15.2 ± 6.1	11.6 ± 4.8	<**0.01**
Hgb, g/dL mean ± SD	13.5 ± 2.1	13.8 ± 1.3	0.89
Sodium, mmol/L mean ± SD	138 ± 4	139 ± 3	0.49
Bicarbonate, mmol/L mean ± SD	25 ± 12	27 ± 15	0.12
Anion Gap, mean ± SD	14 ± 5	13 ± 4	0.25
Glucose, mg/dL^c^ mean ± SD	188 ± 52	162 ± 59	<**0.01**
Creatinine, mg/dL^d^ mean ± SD	1.46 ± 2.99	0.96 ± 0.8	0.29
Troponin I, ng/dL^e^ mean ± SD	0.38 ± 0.72	0.14 ± 0.42	<**0.01**

*Statistically significant values are given in bold (p-value ≤ 0.05)*.

a *No definitive bleeding source found in 10 patients*.

b *Echocardiogram was not performed in 4 patients*.

c *Conversion factor: multiply by 0.0555 to convert glucose from mg/dL to mmol/L*.

d *Conversion factor: multiply by 76.25 to convert creatinine from mg/dL to μmol/L*.

e *Conversion factor: multiply by 1 to convert troponin I from ng/dL to μg/L*.

*SD, standard deviation; IQR, interquartile range; WBC, white blood cell count; Hgb, hemoglobin*.

**Figure 1:**
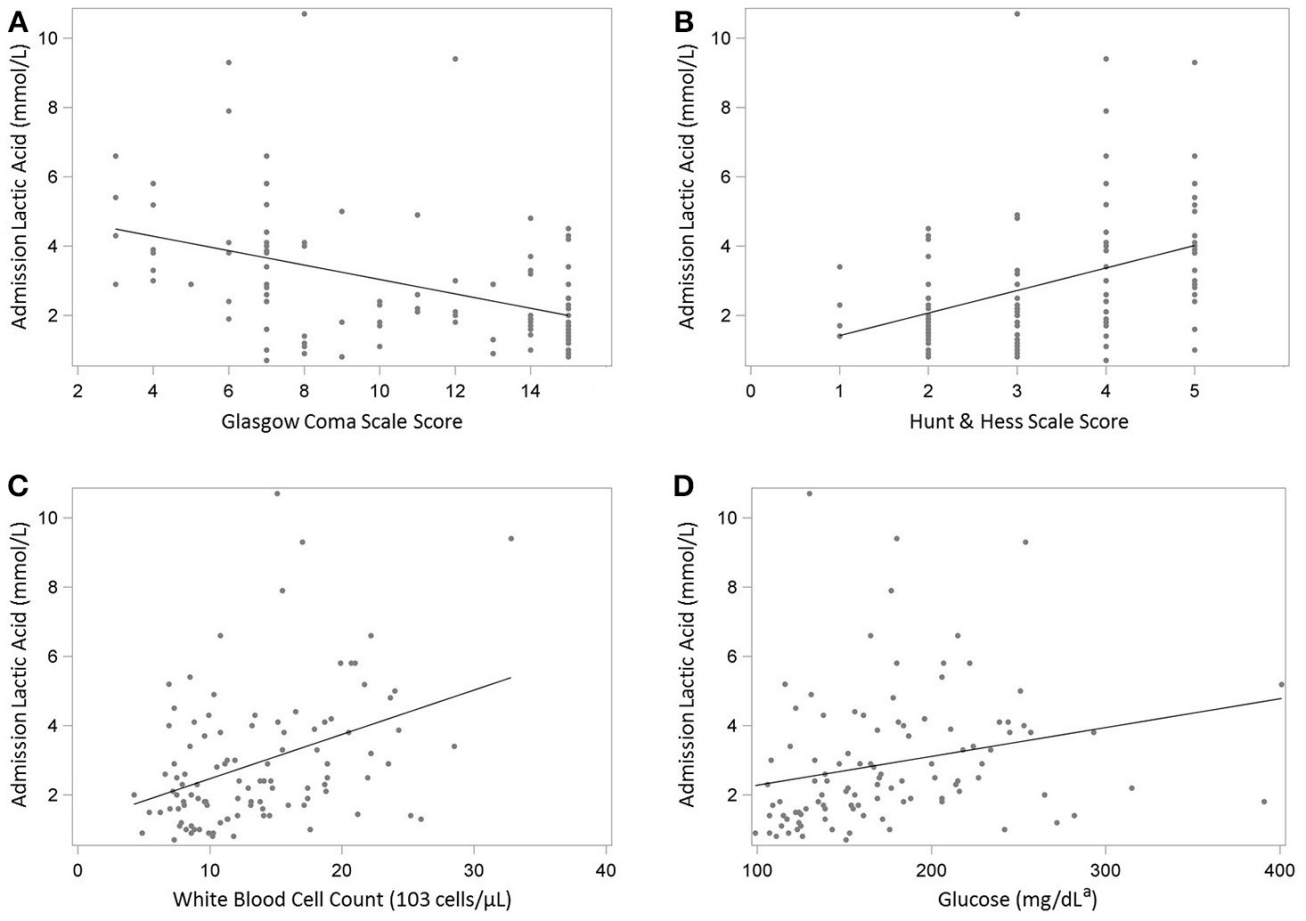
**(A-D)** Scatterplots illustrating the Spearman's correlation between admission serum lactate level and clinical and physiologic markers of disease severity in 105 patients with suspected aneurysmal subarachnoid hemorrhage. **(A)** Glasgow Coma Scale score. **(B)** Hunt & Hess Scale score. **(C)** White blood cell count. **(D)** Glucose. ^a^Conversion factor: multiply by 0.0555 to convert glucose from mg/dL to mmol/L.

In Spearman correlation coefficient analysis (Table 2), LA was positively associated with HH grade (*r* = 0.49), modified Fisher grade (*r* = 0.32), glucose (*r* = 0.41), troponin I (*r* = 0.30), WBC (*r* = 0.36) (all *p* < 0.05), as well as EVD days (*r* = 0.20, *p* = 0.05). LA was negatively associated with GCS (*r* = −0.48) and ventilator-free days (*r* = −0.35). Scatterplots of significant correlations between serum lactate level and clinical and physiologic markers of disease severity are shown in (Figure 1A–D). Panels A and B illustrate the correlation between LA and the patient's clinical grade on presentation. Panels C and D illustrate the positive correlation between lactate and WBC and glucose.

**Table 2:** Spearman correlation between admission serum lactic acid level and patient characteristics and outcomes for 105 suspected aneurysmal subarachnoid hemorrhage patients.

**Variable**	**Spearman correlation coefficient (*r*)**	***p*-value**
Age, years	0.00	0.97
GCS score	−0.48	<**0.01**
Hunt & Hess grade	0.49	<**0.01**
Modified Fisher grade	0.32	<**0.01**
WBC, 10^3^ cells/μL	0.36	<**0.01**
Hemoglobin, g/dL	0.07	0.45
Sodium, mmol/L	0.00	0.99
Bicarbonate, mmol/L	−0.15	0.13
Anion Gap	0.14	0.14
Glucose, mg/dL^a^	0.41	<**0.01**
Creatinine, mg/dL^b^	−0.03	0.73
Troponin I, ng/dL^c^	0.30	<**0.01**
EVD days	0.20	**0.05**
Ventilator-free days	−0.35	<**0.01**
ICU days	0.06	0.54
Hospital Days	0.03	0.74

*Coefficient (r) values > 0 indicate a positive association; values < 0 indicate a negative association. Statistically significant values are given in bold (p-value ≤ 0.05)*.

a *Conversion factor: multiply by 0.0555 to convert glucose from mg/dL to mmol/L*.

b *Conversion factor: multiply by 76.25 to convert creatinine from mg/dL to μmol/L*.

c *Conversion factor: multiply by 1 to convert troponin I from ng/dL to μg/L*.

*GCS, Glasgow Coma Score; WBC, white blood cell count; EVD, external ventricular drain; ICU, intensive care unit*.

Hospital outcomes are displayed in Table 3. EVD placement occurred more frequently in the elevated LA group (*p* < 0.01). Patients with elevated LA had significantly fewer ventilator-free days, higher rates of in-patient mortality, and were less likely to be discharged home. No difference in EVD days, ICU days, hospital days, or incidence of vasospasm or DCI were observed. When adjusting for age, GCS, HH score, and DCI, the adjusted odds of death (odds ratio, 0.97; confidence interval, 0.79–1.2; *p* = 0.80) and discharge home (odds ratio, 1.0; confidence interval, 08–1.26; *p* = 0.97) was not significantly different between groups (Table 4).

**Table 3:** Univariate analysis of in-hospital outcomes for 105 suspected aneurysmal subarachnoid hemorrhage patients.

**Outcome**	**Lactate elevated (*n* = 56) No. (%)**	**Lactate normal (*n* = 49) No. (%)**	***p*-value**
EVD			<**0.01**
Yes	53 (95)	37 (76)	
No	3 (5)	12 (24)	
EVD days, mean ± SD	11.6 ± 7.2	10.6 ± 10.0	0.17
Aneurysm Procedure			
Clip/Wrap	18 (32)	21 (43)	0.59
Coil	24 (43)	18 (37)	
None	14 (25)	10 (20)	
Vasospasm			0.92
Yes	28 (50)	25 (47.2)	
No	28 (50)	24 (46.1)	
DCI			0.28
Yes	11 (20)	14 (29)	
No	45 (80)	35 (71)	
Hosp Mortality			<**0.01**
Yes	22 (39)	6 (12)	
No	34 (61)	43 (88)	
Discharge Home			<**0.01**
Yes	16 (29)	27 (55)	
No	40 (71)	22 (45)	
Ventilator-free days, mean ± SD	11.8 ± 12.3	19.6 ± 10.8	<**0.01**
ICU days, mean ± SD	13.7 ± 7.1	14.3 ± 8.6	0.81
Hospital Days, mean ± SD	17.8 ± 13.6	18.7 ± 12.3	0.94

*Statistically significant values are given in bold (p-value ≤ 0.05). EVD, external ventricular drain; SD: standard deviation; DCI, delayed cerebralischemia; ICU, intensive care unit*.

**Table 4:** The adjusted odds of inpatient mortality and of being discharged home, associated with admission lactic acid level in 105 suspected aneurysmal subarachnoid hemorrhage patients.

**Outcome**	**Adjusted odds ratio**	**95% CI**	***p*-value**
Death	0.97	0.79-1.20	0.80
Discharge Home	1.00	0.80-1.26	0.97

*CI: confidence interval*.

## Discussion

Physiologic disturbances following aSAH lead to several potential laboratory abnormalities and worse patient outcomes (11). Elevations in serum LA, a marker of metabolic crisis, has been observed in the acute phase of the disease (8, 9, 12); however, its clinical significance remains understudied. We therefore aimed to further characterize LA in this population. To our knowledge, this is the most comprehensive analysis of admission variables and patient outcomes related to peripherally-drawn LA in aSAH.

In this study, 53% of patients presented with elevated serum LA (mean of 2.91 mmol/L). The reason for increased lactate production in the hyperacute phase of aSAH is not well understood. Potential sources are primary brain LA generation during states of hyperglycolysis or cerebral metabolic crisis, vs. acidemia secondary to a systemic process. In our study, strong associations with glucose and WBC count suggest a relation to systemic inflammation and sympathetic overactivity, both of which occur in aSAH (13–15). Each can occur independent of one another (16), with LA potentially resulting from either process. A neurohormonal response might also account for elevations in troponins and higher incidence of WMAs in our cohort. Cardiac complications after aSAH are common (17, 18); however, in our study lactic acidemia occurred in the absence of systolic dysfunction contributing to systemic tissue hypoperfusion. At our institution, the presence of predisposing conditions, such as hypovolemia and infection, and their association with type A lactic acidosis is part of on-going research but was beyond the scope of this study. Although we could not exclude occult infection in this cohort, systemic inflammatory response syndrome after intracranial hemorrhage does not predict positive cultures (19), while nosocomial infections in aSAH occur after days and would not contribute to elevations in LA at the time of admission (20).

There is increasing interest in lactate as a potential marker of neurologic injury severity. In other critical care specialties, serum LA has been used to predict outcomes in several conditions, including sepsis and cardiac injury (21–23). Our results suggest that serum LA at the time of admission is strongly associated with the clinical and radiographic grade of aSAH and correlates well with other physiologic markers of disease severity. Serum lactate is a quantitative and widely available measure that, in conjunction with current grading scales, may be useful in classifying aSAH severity based on physiologic derangements (11). In our study, we additionally found that early serum LA was associated with higher odds of EVD placement and was inversely associated with ventilator-free days. This is consistent with LA being a marker of illness severity; however, further study is needed to determine if LA reliably predicts complications like pulmonary edema or pneumonia (17).

Patients with elevated lactate were more likely to sustain mortality and less likely to be discharged home; however, after adjusting for age, GCS, HH score, and DCI, our results do not provide evidence that admission serum LA independently predicts outcomes in SAH. These findings contrast with three recent retrospective studies that suggest peripherally drawn LA might predict DCI, mortality and poor functional outcomes (8, 9, 12); however, none included other admission variables and short-term outcomes as comprehensively as this study. Selection bias and differences in clinical practice might also account for differences in results.

It is possible to differentiate between “good” and “bad” LA, where cerebral lactate is only considered harmful if accompanied by cerebral oligemia or hypoxemia (5–7). Similarly, serum lactate might be an important clinical target if caused by a reversible systemic process. For example, hypovolemia is common in aSAH (24–26), potentially resulting in Type A lactic acidosis and reduced cerebral perfusion. In our practice, if admission lactic acidemia is accompanied by clinical hypovolemia, patients are aggressively fluid-resuscitated in attempts to reduce secondary brain injury. Ultrasonography of the inferior vena cava is a rapid bedside tool we additionally use to aid in predicting fluid-responsiveness in aSAH (26), helping guide acute hemodynamic management. LA could also be used to measure the effectiveness of treatments meant to reduce systemic inflammation or sympathetic overactivity. In this context, persistent lactic acidemia or lactate clearance may be more useful measurement (12).

This study has other limitations. A larger cohort is needed to detect the true relationship between lactate and clinical outcomes. The small sample size also did not allow us to adjust for imbalances in baseline characteristics. Importantly, as a retrospective study, we were unable to control for all confounding factors. Although we have incorporated LA testing into our routine admission orders, patients with poorer clinical and radiographic grades of SAH, or those with systemic inflammatory response syndrome may have been more likely to have LA tested and receive more intensive hemodynamic management. Retrospective analysis is also susceptible to missing information. Admission blood pH was missing in a several patients, particularly those who were not intubated or went for early surgery and was not included in our analysis. As a result, the impact of observed elevated admission LA on acid-base balance remains unknown. Although there were few other absent radiographic and laboratory values in our dataset, long-term functional outcomes were not analyzed, but considered out of the scope of this study. Lastly, direct causal relationships cannot be established.

The results of this investigation are hypothesis-generating, with future studies needed to characterize the clinical significance of this recently described finding. Correlating admission serum lactate with multimodal markers of cerebral metabolism would be useful in determining the primary source of LA generation, with additional opportunities to investigate the relationship between serum LA with fluid status in aSAH.

## Conclusions

Early lactic acidemia is common in aSAH patients and is associated with the clinical and radiographic grade of hemorrhage. The primary source of LA is still undetermined, but likely multifactorial. Peripherally-drawn LA is not associated with the development of vasospasm, DCI, mortality or discharge disposition when controlled for disease severity; however, its clinical utility in aSAH is still unknown. Admission LA levels may help clinicians identify potentially reversible pathophysiologic processes, including hypovolemia. Future studies should further determine sources of LA production and investigate its role as a therapeutic target to guide early management.

## Ethics statement

This study was carried out in accordance with the recommendations and approval of the USC Health Sciences Institutional Review Board. The need for informed consent was waived due to the retrospective nature of the study with no more than minimal risk to the patient.

## Author contributions

RP and BE conceived the hypothesis and designed the study. BE directed the study. RP was the primary author of the manuscript with input from all authors. SC and LZ performed statistical analysis and wrote sections of the manuscript. RP and BE were involved in critical manuscript revisions. All authors read and approved the submitted version.

### Conflict of interest statement

The authors declare that the research was conducted in the absence of any commercial or financial relationships that could be construed as a potential conflict of interest.
